# Influence of the Adequacy of the Prenatal Care Utilization Index on Small-For-Gestational-Age Infants and Preterm Births in the United States

**DOI:** 10.3390/jcm8060838

**Published:** 2019-06-12

**Authors:** Dayeon Shin, Won O. Song

**Affiliations:** 1 Department of Food and Nutrition, Inha University, Incheon 22212, Korea; dyshin@inha.ac.kr; 2 Department of Food Science and Human Nutrition, Michigan State University, East Lansing, MI 48824, USA

**Keywords:** Pregnancy Risk Assessment Monitoring System (PRAMS), Adequacy of Prenatal Care Utilization (APNCU) index, small-for-gestational-age (SGA), preterm birth

## Abstract

Little is known about the associations of Adequacy of Prenatal Care Utilization (APNCU) index with small-for-gestational-age (SGA) infants and preterm births. This study investigated the association between the Adequacy of Prenatal Care Utilization (APNCU) index in relation to small-for-gestational-age (SGA) infants and preterm births. We used data from 212,050 pregnant women from the Pregnancy Risk Assessment Monitoring System (PRAMS) between 2004 and 2011. Multivariable logistic regression analyses were performed to examine the effect of the APNCU index on SGA infants and preterm births after controlling for maternal sociodemographic factors. Women who received adequate-plus prenatal care in reference to adequate prenatal care had increased odds for delivering SGA infants (adjusted odds ratio (AOR) = 1.08, 95% confidence interval (CI) = 1.03–1.15). Women with 9–11 prenatal care visits had increased odds of delivering SGA infants (AOR = 1.07, 95% CI = 1.02–1.14) compared to those with more than 12 visits. Among the four APNCU index categories, the highest rate of preterm births was observed in the adequate-plus group. Compared to those with adequate prenatal care, women who received adequate-plus prenatal care had increased odds of preterm birth (AOR = 1.69, 95% CI = 1.55–1.84). Compared to those with more than 12 visits, women with fewer than eight prenatal care visits had increased odds of preterm birth (AOR = 1.29, 95% CI = 1.13–1.48). In conclusion, women in the adequate-plus APNCU index category were more likely to deliver SGA infants and to have preterm births compared to those in the adequate APNCU index category. Women in the U.S. with high-risk pregnancies were prone to receiving adequate-plus prenatal care. Future prospective studies are warranted to investigate the influence of APNCU index in relation to pregnancy and birth outcomes.

## 1. Introduction

Preterm birth is the most frequent cause of infant and neonatal death in the U.S. [1] and is also the most important factor influencing an infant’s subsequent health and survival [2]. Compared to full-term infants (37–41 weeks of gestation), preterm infants (<37 weeks of gestation) have a wide variety of health and developmental problems, including long-term cognitive, behavioral, social, emotional, and neurodevelopmental difficulties [3]. Low birth weight and small-for-gestational-age (SGA) infants are the next most common causes of infant death [1]. Also, low birth weight and SGA are associated with poor neurocognitive development among infants [4]. For these reason, Healthy People 2020, the health objectives for the nation, includes the goal of a reduction of low birth weight rate from a baseline of 8.2% to 7.8% of live births by 2020 [5].

An important approach to reducing the risk of preterm birth and SGA infants is adequate prenatal care [6,7]. Prenatal care is a frequently used health service that may reduce the incidence of perinatal morbidity and mortality by treating medical conditions, identifying and reducing potential risks, and helping women to address behavioral factors that contribute to poor outcomes [8]. Studies analyzing trends in prenatal care and birth outcomes have used a number of methods to assess the adequacy of prenatal care [9]. One of the more recently developed methods, the Adequacy of Prenatal Care Utilization (APNCU) index, is an improvement on the Kessner Index from the Institute of Medicine, which considers only the trimester of initiation of prenatal care and the number of prenatal visits [10]. The APNCU index is used for precise and comprehensive measurement of prenatal care [11].

The beneficial effect of prenatal care utilization indicated by the APNCU index was a reduced risk of preterm birth or SGA [6,12,13,14,15], whereas no beneficial effects of prenatal care were shown in the prevention of adverse birth outcomes [16,17,18,19]. There is still significant debate in the U.S. regarding the effectiveness of prenatal care in reducing SGA and preterm-birth pregnant women. The objectives of this study were to determine the rate of prenatal care utilization among pregnant women in the U.S. and to determine the association of the adequacy of prenatal care utilization with SGA and preterm birth. We hypothesized that the adequacy of prenatal care utilization is associated with the risk of SGA and preterm birth in U.S. pregnant women.

## 2. Materials and Methods

### 2.1. Study Populations

The present study used data from the Pregnancy Risk Assessment Monitoring System (PRAMS). The PRAMS is an ongoing surveillance project from the Centers for Disease Control and Prevention (CDC) and state health departments of 40 U.S. states and New York City. The most recent dataset that was attainable at the beginning of this project was from 2004 to 2011, including phases 5 (2004–2008) and 6 (2009–2011). The PRAMS sample is chosen from among all women with recent live births; therefore, findings can be applied to the participating state′s entire population of women who have recently delivered live-born infants. The PRAMS provides state-specific data, and also allows for comparisons among participating states because the same data collection methods are used in all states. The PRAMS, which collects data from the state birth certificate files, is a stratified systematic sample of 100–300 new mothers who have delivered live-born infants in the preceding 2–4 months. A self-administered questionnaire is mailed to each mother. If the mother fails to respond a second, and usually a third, questionnaire is mailed to each mother. If the mother does not respond to the mailings, telephone interviews are used for follow-up. Each completed questionnaire is then linked to information from the state’s birth certificate file. The birth certificate files include information on total gestational weight gain and SGA infants. A self-administered questionnaire was mailed to each mother to obtain information on preterm birth. As the survey is conducted several months after delivery, recall bias is possible regarding the mothers’ observations or experiences [20]. However, recall bias is of minimal concern for risk factors related to maternal or neonatal morbidity [21].

The initial PRAMS 2004–2011 cohort included 313,735 women from Michigan. After excluding women with missing data on APNCU (*N* = 11,445), the number of prenatal care visits (*N* = 1366), time of the first prenatal care initiation (*N* = 6565), starting prenatal care in the first trimester (*N* = 7), previous history of preterm birth (*N* = 4785), SGA infants (*N* = 22,336), pre-pregnancy body mass index (BMI) (*N* = 14,181), gestational weight gain (*N* = 17,460), and maternal sociodemographic characteristic variables (*N* = 23,540), the final analytic sample size for the present study was 212,050 women.

### 2.2. Exposure Variables

The APNCU index developed by Kotelchuck determines the adequacy of prenatal care utilization based on two parts: the month in which prenatal care is initiated and the number of visits from initiation of care until delivery and then categorized into four: “Inadequate” care is defined as either starting prenatal care after the 4th month of pregnancy or receiving less than 50% of expected visits based on the schedule of prenatal care visits recommended by American College of Obstetricians and Gynecologists (ACOG). “Intermediate” care is care begun by month 4 and with 50–79% of expected visits received; “adequate” care is that begun by month 4 and with 80–109% of expected visits received; “adequate plus” care is begun by month 4 and with 110% or more of expected visits received [10]. The expected number of prenatal visits was calculated from the month of initiation of prenatal care and gestational age at birth, based on the schedule of prenatal care visits recommended by the American College of Obstetrics and Gynecology (ACOG). The ACOG recommends one visit every four weeks for the first 28 weeks, five times for 32 weeks, six times for 36 weeks, and 7–11 times for 37–41 weeks of pregnancy [22]. Consequently, the ratio of observed number to expected number of visits was calculated and used in categorizing women into four different groups: inadequate, intermediate, adequate, and adequate-plus utilization of prenatal care services. Inadequate utilization is defined as either starting prenatal care after the 4th month of pregnancy or receiving fewer than 50% of the expected visits based on the schedule of prenatal care visits recommended by the ACOG. Intermediate care is care begun by month 4 and with between 50% and 79% of the expected visits received; adequate care is that begun by month 4 and with 80–109% of the expected visits received; adequate-plus care is begun by month 4 and with 110% or more of the expected visits received.

The initiation of prenatal care in the first trimester was categorized as yes, no, or no prenatal care. The number of prenatal care visits was categorized into less than eight visits, 9–11 visits, or more than 12 visits.

### 2.3. Outcome Variables

If the birth certificate indicated that the infant’s birth weight was below the 10th percentile for the gestational age, the mother was determined to have experienced the outcome of SGA. From the PRAMS questionnaire, if the infant was born at <37 completed weeks of gestation, the mother was considered to have had a preterm birth.

### 2.4. Covariates

Covariates in the study were maternal age in three groups (≤24, 25–34, or ≥35 years). race/ethnicity consisted of non-Hispanic white, non-Hispanic black, Hispanic, and other non-Hispanic race/ethnicities, maternal education clustered (<high school, high school diploma, or more than high school), annual household income classified into five categories (<$15,000, $15,000–35,000, $35,000–50,000, or ≥$50,000). Marital status was divided into two groups (married or other). Gestational age at birth was categorized into five groups (≤27, 28–33, 34–36, 37–42, or ≥43 weeks). Women, infants, and children (WIC) status during pregnancy was divided into two groups (yes or no). Smoking status was divided into two groups (yes or no). Previous preterm birth was categorized into two groups (yes or no). Parity number was categorized into five groups (0, 1, 2, 3–5, or ≥6).

### 2.5. Statistical Analyses

The participants’ characteristics were described using weighted frequency distributions and adjusted for survey sampling. Tests of associations between the APNCU index and maternal characteristics were performed using chi-squared statistics. Multivariable logistic regression was used to examine the relationship between the adequacy of prenatal care and SGA infants or preterm births as an outcome after controlling for pre-pregnancy BMI, gestational weight gain, maternal age, race, education level, income level, marital status, gestational weeks, WIC participation during pregnancy, smoking status during pregnancy, and previous history of preterm birth. The independent variables of interest were the APNCU index (adequate-plus, adequate, intermediate, and inadequate utilization of prenatal care), timing of the initiation of prenatal care (start prenatal care in the first trimester, start prenatal care in the second or third trimester, or none) and the number of prenatal care visits (≥12, 9–11, or ≤8).

To obtain findings applicable to all women in the US, sample weights were applied to account for selection and response probabilities of the survey design. SAS 9.4 (SAS Institute, Cary, NC, USA) was used for statistical analyses. Approval for data use was obtained from the PRAMS Working Group at the CDC for the data analysis.

## 3. Results

Table 1 presents the distributions of maternal characteristics by SGA and preterm births. Pre-pregnancy BMI, the adequacy of gestational weight gain, maternal age, education, annual household income, marital status, gestational age at birth, WIC participation during pregnancy, smoking status, previous live birth number, timing of initiation of prenatal care, and number of prenatal care visit all significantly differed by the status of SGA and preterm birth, respectively (all *p* < 0.05). Maternal race only differed by the status of preterm birth.

Table 2 shows the characteristics of our study population by APNCU index category (inadequate, intermediate, adequate, or adequate-plus). Overall, 10.5% of women received inadequate prenatal care, 13.7% received intermediate prenatal care, 46.9% received adequate prenatal care, and 28.9% received adequate-plus prenatal care according to the APNCU index. Differences in pre-pregnancy BMI, gestational weight gain, maternal age, race, education, annual income, marital status, gestational weeks, WIC participation during pregnancy, smoking status during pregnancy, and parity across APNCU index categories were all significant (*p* < 0.0001).

Table 3 shows the distributions of women with SGA infants and preterm births by APNCU index categories. The distributions of SGA infants and preterm births differed significantly by each APNCU category, respectively (*p* < 0.0001). Among the four APNCU categories, the highest rate of SGA infant was observed in the adequate group (43.7%), whereas the highest rate of preterm birth was observed in the adequate-plus group (41.0%).

Compared to those who had received adequate prenatal care, women who received adequate-plus prenatal care had higher odds of delivering SGA infants (adjusted odds ratio (AOR) = 1.08; 95% CI = 1.03–1.15). Compared to those who had received adequate prenatal care, women who received adequate-plus prenatal care had higher odds for preterm birth (AOR = 1.69; 95% CI = 1.55–1.84) (Table 4).

Women who did not receive any prenatal care during pregnancy had increased odds of delivering SGA infants compared to those in women who started in the first trimester (AOR = 1.37, 95% CI = 1.03–1.84). Women who started prenatal care in the second or third trimester had lower odds of preterm births than that in women who started prenatal care in the first trimester (AOR = 0.89, 95% CI = 0.81–0.99) (Table 5).

Women who received 9–11 prenatal care visits had increased odds of delivering SGA infants compared to those in women who had more than 12 prenatal care visits (AOR = 1.07, 95% CI = 1.02–1.14). Women who received fewer than eight prenatal care visits had increased odds for preterm birth compared to those in women with more than 12 prenatal care visits (AOR = 1.29, 95% CI = 1.13–1.48) (Table 6).

## 4. Discussion

Our study findings indicated that the effect of inadequate utilization of prenatal care on the risk of SGA birth was not statistically significant, which is in agreement with previous findings [23]. However, inadequate utilization of prenatal care indicated by the APNCU index was reportedly associated with an increased risk for SGA infants in a representative U.S. population [13,24]. This may be due to the fact that those with inadequate utilization of prenatal care were disproportionately mothers under 15 years of age and multiparous women. However, the demographics of pre-pregnancy BMI, maternal age, race, education, and income across the categories of APNCU index categories, as shown in Table 1, showed even distributions.

The results of the present study showed that women who did not receive any prenatal care compared to those women who started prenatal care in the first trimester of pregnancy had increased risks of delivering SGA infants. In addition, women who had 9–11 prenatal care visits, had increased risks for delivering SGA infants compared to those in women with more than 12 prenatal care visits. These results parallel previous findings that the rates of SGA declined with increasing numbers of prenatal care visits [9]. According to previous studies, prenatal care is also beneficial for pregnant women for the diagnosis and treatment of maternal genital tract [25], and HIV infections [26] or for the imitation of exclusive breastfeeding [27].

In the present study, women in the adequate-plus utilization of prenatal care category were at an increased risk for preterm births compared to those in the adequate utilization of prenatal care category. This may be due to the fact that a shorter gestational age implies a lower number of expected visits, which yields a small denominator in the observed/expected ratio of prenatal care visits [9]. As a result, the observed/expected ratios may exceed 100% and may cause misleading results indicating that women grouped in the adequate-plus category are most likely to have a preterm birth. Thus, the APNCU index yielded results indicating that those women categorized in the highest resource utilization category were most likely to experience preterm births, as confirmed in previous findings [9,24]. Our results also indicated that women in the adequate-plus category had the highest number of gestational-age births (at less than 37 weeks) (41.0%) compared to that in women in the inadequate (11.7%), intermediate (10.7%), and adequate (36.7%) APNCU groups. It has been previously suggested that the adequate-plus group includes disproportionately more identified high-risk pregnancies that required more prenatal visits and subsequent interventions [7,10]. Contrary to our findings, among U.S. [6] and Canadian pregnant women [8], the preterm birth rate was significantly higher in the “presence of prenatal care” group compared to that in the “absence of prenatal care” group. However, in that study, prenatal care was considered to be present if there was at least one prenatal visit during the course of pregnancy [6]. The contradictory findings may be due to the definition of the presence of prenatal care, which is different from that in the APNCU index, which considers the month of initiation of prenatal care as well as the total number of prenatal visits.

This study has several limitations. A limitation of the APNCU index is the gestational age bias [9,24]. Gestational age affects categorization within the APNCU index and could have a greater impact on preterm births. Short gestation may result in delivery before the opportunity to initiate care or misclassification into the adequate-plus category, as fewer visits are recommended in early pregnancy and 110% utilization could be met with only one extra visit [24]. Our finding of a 1.69-fold increase in the number of preterm births in the adequate-plus group compared to that in the adequate group may reflect this bias; thus, caution is necessary for the interpretation of the APNCU index in relation to preterm births. Additionally, health insurance information was not considered in assessing the relationship between prenatal care and birth outcomes, although a lack of health insurance is an important risk factor for inadequate prenatal care [28].

Although the APNCU index is a widely considered standard for estimating the adequacy of prenatal care utilization, some researchers [9] reported shortcomings of the index such as a young gestational age implies fewer number of expected visits and, thus, results in the observed/expected ratios often exceeding 100%. Consequently, the authors concluded that the APNCU index yields misleading results indicating that women group in the adequate plus category are most likely to deliver low birth weight infants. Limitations in the definitions and measurement of prenatal care may generate these results, which can also be applied in our study.

Strengths of this study are that PRAMS is a population-based study with the overall response rate of over 70%. The extensive information on maternal sociodemographic and lifestyle factors could be matched with state birth records and, thus, a number of important confounders could be controlled in the present study. However, this study may have several limitations. Due to the retrospective cross-sectional study design, a cause-effect relationship cannot be established. Mothers who were surveyed 2–4 months postpartum could have had some recall bias with memory lapse. Additionally, medically-induced preterm births could not be distinguished from spontaneous preterm births in our study.

## 5. Conclusions

In conclusion, women in the adequate plus APNCU index category are most likely to deliver SGA infants and preterm birth. Fewer numbers of prenatal visits are associated with higher rates of SGA infants and preterm birth. We conclude that women with high-risk pregnancy are prone to receive adequate plus prenatal care in the U.S.

## Figures and Tables

**Table 1:** Maternal sociodemographic characteristics by small-for-gestational-age (SGA) and preterm births.

	SGA (*n* = 35,137)		Non SGA (*n* = 176,913)			Preterm Birth (*n* = 52,602)		Non Preterm Birth (*n* = 159,448)		
	*n*	Wt′d %	*n*	Wt′d %	*p* Value	*n*	Wt′d %	*n*	Wt′d %	*p* Value ^++^
Pre-pregnancy BMI ^1^										
Underweight	3091	8.1	8310	4.3	<0.0001	3773	6.1	7628	4.3	<0.0001
Normal	18,981	56.5	90,417	52.0		26,570	50.2	82,828	52.9	
Overweight	7107	19.9	42,622	24.1		11,846	23.3	37,883	23.8	
Obese	5958	15.4	35,564	19.6		10,413	20.4	31,109	18.9	
Gestational weight gain ^2^										
Inadequate	11,139	28.7	36,438	17.4	<0.0001	15,599	22.3	31,978	17.5	<0.0001
Adequate	11,027	32.1	50,988	28.6		15,259	28.6	46,756	29.0	
Excessive	12,971	39.2	89,487	54.0		21,744	49.1	80,714	53.5	
Maternal age (y)										
≤24	13,698	38.7	56,851	30.3	<0.0001	21,556	38.4	48,993	29.4	<0.0001
25–34	16,375	48.1	92,552	54.9		24,750	50.4	84,177	55.2	
≥35	5064	13.2	27,510	14.8		6296	11.2	26,278	15.5	
Maternal race										
Non-Hispanic White	22,025	65.7	101,487	65.4	0.1082	31,367	64.7	92,145	65.6	<0.0001
Non-Hispanic Black	5362	12.8	26,490	13.3		9766	16.3	22,086	12.5	
Hispanic	4051	14.0	22,660	14.3		6284	13.9	20,427	14.4	
Other non-Hispanic	3699	7.6	26,276	7.0		5185	5.1	24,790	7.5	
Maternal education										
<High school	6029	17.5	24,170	13.6	<0.0001	9760	18.0	20,439	13.0	<0.0001
High school diploma	11,314	31.0	49,644	26.8		17,370	32.0	43,588	26.0	
Some college	8830	24.5	46,779	26.0		14,417	27.8	41,192	25.4	
≥College	8964	27.0	56,320	33.6		11,055	22.2	54,229	35.5	
Annual household income										
Less than $15,000	12,978	35.7	50,734	26.4	<0.0001	20,011	35.1	43,701	25.5	<0.0001
$15,000–$34,999	8737	24.4	42,795	23.5		13,754	26.2	37,778	23.0	
$35,000-$50,000	3591	9.9	19,815	10.9		5441	10.8	17,965	10.8	
≥$50,000	9831	30.0	63,569	39.2		13,396	28.0	60,004	40.8	
Marital status										
Married	20,130	56.2	114,569	65.8	<0.0001	29,606	57.5	105,093	66.7	<0.0001
Other	15,007	43.8	62,344	34.2		22,996	42.5	54,355	33.3	
Gestational age at birth (weeks)										
≤27	473	0.5	4152	0.4	0.023	2749	1.2	1876	0.2	<0.0001
28–33	1694	1.5	11,167	1.4		6920	4.1	5941	0.8	
34–36	5203	6.3	18,628	5.6		13,025	16.4	10,806	3.1	
37–42	27,737	91.5	142,815	92.5		29,875	78.1	140,677	95.8	
≥43	30	0.1	151	0.1		33	0.1	148	0.1	
WIC during pregnancy										
Yes	17,371	51.3	99,930	59.0	<.0001	24,340	48.9	92,961	60.5	<.0001
No	17,766	48.7	76,983	41.0		28,262	51.1	66,487	39.5	
Smoking status										
Yes	7793	19.8	20,047	9.7	<0.0001	10,025	16.3	17,815	9.3	<0.0001
No	27,344	80.2	156,866	90.3		42,577	83.7	141,633	90.7	
Previous live birth number										
0	18,668	53.2	73,933	40.8	<0.0001	19,945	33.4	72,656	44.0	<0.0001
1	9050	26.5	56,336	33.2		16,335	34.6	49,051	32.1	
2	4575	12.8	28,180	16.0		9370	18.6	23,385	15.0	
3–5	2637	6.9	17,194	9.2		6472	12.6	13,359	8.2	
6+	207	0.5	1270	0.7		480	0.8	997	0.6	
Timing of initiation of prenatal care										
1st trimester	27,971	79.1	144,823	82.4	<0.0001	42,285	80.8	130,509	82.4	<0.0001
2nd or 3rd trimester	6881	19.9	30,713	17.0		9860	18.6	27,734	16.9	
None	285	1.0	1377	0.6		457	0.7	1205	0.6	
Number of prenatal care visits										
≤8 times	8256	20.3	38,701	17.4	<0.0001	15,783	21.8	31,174	16.6	<0.0001
9–11 times	11,524	32.6	56,201	32.1		15,687	30.7	52,038	32.5	
≥12 times	15,357	47.1	82,011	50.5		21,132	47.5	76,236	50.9	

^++^*p* value: Chi-squared tests for differences in SGA and preterm births by each sociodemographic variable. Weighted (Wt’d) % accounted for the survey sampling design and coverage. The weighted percentages may not sum to 100 due to rounding. ^1^ Pre-pregnancy body mass index (BMI) (kg/m^2^) categories according to the World Health Organization: underweight (<18.5), normal weight (18.5–24.9), overweight (25–29.9), and obese (≥30) groups. ^2^ Gestational weight gain was divided into inadequate, adequate, and excessive groups according to Institute of Medicine’s 2009 guidelines. WIC: Women, infants and children.

**Table 2:** Maternal sociodemographic characteristics across Adequacy of Prenatal Care Utilization (APNCU) index categories.

	APNCU Index Category ^1^								
	Inadequate		Intermediate		Adequate		Adequate Plus		*p* Value
	(*N* = 22,487; 10.5%)		(*N* = 27,147; 13.7%)		(*N* = 89,804; 46.9%)		(*N* = 72,612; 28.9%)		
	*n*	Wt′d %	*n*	Wt′d %	*n*	Wt′d %	*n*	Wt′d %	
Pre-pregnancy BMI ^2^									
Underweight	1588	13.8	1388	13	4289	42.8	4136	30.4	<0.0001
Normal	11,416	10.2	14,649	14.2	48,185	48.3	35,148	27.2	
Overweight	5185	10.4	6446	14	21,165	46.9	16,933	28.7	
Obese	4298	10.7	4664	11.8	16,165	44	16,395	33.4	
Gestational weight gain ^3^									
Inadequate	6884	15.1	6093	14.1	16,971	41.7	17,629	29.1	<0.0001
Adequate	6033	9.9	8125	14	26,949	48	20,908	28.1	
Excessive	9570	9.3	12,929	13.3	45,884	48.1	34,075	29.2	
Maternal age (y)									
≤24	10,968	16.2	9119	14	27,162	42.5	23,300	27.3	<0.0001
25–34	9026	8.2	13,871	13.4	48,841	49.2	37,189	29.1	
≥35	2493	7.3	4157	13.8	13,801	47.6	12,123	31.4	
Maternal race									
Non-Hispanic White	9556	7.9	14,320	13	54,642	49.3	44,994	29.9	<0.0001
Non-Hispanic Black	5098	17.4	3988	14.5	11,207	39	11,559	29.1	
Hispanic	3994	15.8	4013	15.5	10,858	43.5	7846	25.1	
Other non-Hispanic	3839	12	4826	15	13,097	46.5	8213	26.5	
Maternal education									
<High school	6197	21.5	4208	15.2	10,501	38.5	9293	24.8	<0.0001
High school diploma	7989	13.2	7691	13.5	24,052	44	21,226	29.2	
Some college	5172	9.2	7043	13.3	23,769	47.3	19,625	30.3	
≥College	3129	4.7	8205	13.5	31,482	52.5	22,468	29.2	
Annual household income									
Less than $15,000	11,654	19.1	8410	14.2	22,819	39.8	20,829	26.8	<0.0001
$15,000–$34,999	5954	12.2	6592	13.7	21,273	45.2	17,713	28.9	
$35,000–$50,000	1687	7.3	2929	13	10,618	49.5	8172	30.2	
≥$50,000	3192	4.3	9216	13.4	35,094	52.2	25,898	30	
Marital status									
Married	9505	6.9	17,205	13.6	61,061	49.8	46,928	29.7	<0.0001
Other	12,982	17.2	9942	13.9	28,743	41.4	25,684	27.4	
Gestational age at birth (weeks)									
≤27	471	10.2	272	6.5	844	18.5	3038	64.9	<0.0001
28–33	1392	12.5	531	4.3	2141	15.4	8797	67.9	
34–36	2653	10.4	1657	7	4207	17.8	15,314	64.7	
37–42	17,934	10.5	24,610	14.2	82,559	49.3	45,449	25.9	
≥43	37	25.3	77	31.9	53	35.2	14	7.7	
WIC during pregnancy									
Yes	13,601	15.2	12,348	13.9	36,243	42.4	32,557	28.6	<0.0001
No	8886	7.2	14,799	13.5	53,561	50.1	40,055	29.1	
Smoking status									
Yes	4839	16.2	3509	12.9	9765	40.7	9727	30.1	<0.0001
No	17,648	9.9	23,638	13.8	80,039	47.6	62,885	28.7	
Previous live birth number									
0	8890	9.7	11,251	13.4	39,078	47.2	33,382	29.7	<0.0001
1	6208	9.3	8509	13.6	28,921	48.4	21,748	28.7	
2	3698	11.1	4393	13.9	13,792	46.5	10,872	28.5	
3-5	3298	16.8	2734	14.3	7573	41.9	6226	27.1	
6+	393	27	260	21.2	440	32.5	384	19.2	
Timing of initiation of prenatal care									
1st trimester	10,735	6.2	21,882	13.6	77,174	49.4	63,003	30.8	<0.0001
2nd or 3rd trimester	10,978	29.8	5089	14.1	12,226	35.6	9301	20.5	
None	774	48.4	176	9	404	27	308	15.6	
Number of prenatal care visits									
≤8 times	16,654	42.4	15,638	41.5	8764	12.4	5901	3.7	<0.0001
9–11 times	3985	6.5	11,460	19.7	36,469	60	15,811	13.8	
≥12 times	1848	1.9	49	0	44,571	50.6	50,900	47.4	

*p* value: Chi-squared tests for differences in APNCU by each sociodemographic variable. Weighted (Wt’d) % accounted for the survey sampling design and coverage. The weighted percentages may not sum to 100 due to rounding. ^1^ The APNCU index comprises two parts: the month in which prenatal care is initiated and the number of visits from the initiation of care until delivery. Inadequate utilization is defined as either starting prenatal care after the 4th month of pregnancy or receiving fewer than 50% of the expected visits based on the schedule for prenatal care visits recommended by the American College of Obstetricians and Gynecologists (ACOG). Intermediate care is care begun by month 4 and with 50–79% of the expected visits received; adequate care is that begun by month 4 and with 80–109% of the expected visits received; adequate-plus care is begun by month 4 and with 110% or more of the expected visits received. ^2^ Pre-pregnancy body mass index (BMI) (kg/m^2^) categories according to the World Health Organization: underweight (<18.5), normal weight (18.5–24.9), overweight (25–29.9), and obese (≥30) groups. ^3^ Gestational weight gain was divided into inadequate, adequate, and excessive groups according to Institute of Medicine’s 2009 guidelines. WIC: Women, infants and children.

**Table 3:** Distributions of small-for-gestational-age (SGA) and preterm births by Adequate Prenatal Care Utilization (APNCU) index categories.

		^1^ APNCU Index Category								
		Inadequate		Intermediate		Adequate		Adequate Plus		*p* Value
		*n*	Wt′d %	*n*	Wt′d %	*n*	Wt′d %	*n*	Wt′d %	
SGA	Yes	4111	12.2	4194	14.9	13,000	43.7	13,832	29.2	<0.0001
	No	18,376	10.4	22,953	13.5	76,804	47.2	58,780	28.9	
Preterm Birth	Yes	6004	11.7	4999	10.7	16,404	36.7	25,195	41.0	<0.0001
	No	16,483	10.3	22,148	14.4	73,400	49.3	47,417	26.1	

*p* value: Chi-squared tests for differences in APNCU by SGA and preterm birth. Weighted (Wt’d) % accounted for the survey sampling design and coverage. The weighted percentages may not sum to 100 due to rounding. ^1^ The APNCU index comprises two parts: the month in which prenatal care is initiated and the number of visits from the initiation of care until delivery. Inadequate utilization is defined as either starting prenatal care after the 4th month of pregnancy or receiving fewer than 50% of the expected visits based on the schedule for prenatal care visits recommended by the American College of Obstetricians and Gynecologists (ACOG). Intermediate care is care begun by month 4 and with 50–79% of expected visits received; adequate care is that begun by month 4 and with 80–109% of the expected visits received; adequate-plus care is begun by month 4 and with 110% or more of the expected visits received.

**Table 4:** Associations of Adequacy of Prenatal Care Utilization (APNCU) index categories with small-for-gestational-age (SGA) infants and preterm births.

	^1^ APNCU Index Category												
	Adequate	Inadequate				Intermediate				Adequate Plus			
	OR	AOR	95% CI		*p* Value	AOR	95% CI		*p* Value	AOR	95% CI		*p* Value
SGA ^†^	1.00 (Ref.)	1.00	0.91	1.09	0.94	1.10	1.01	1.20	0.03	1.08	1.03	1.15	0.005
Preterm Birth ^‡^	1.00 (Ref.)	1.01	0.88	1.16	0.90	0.89	0.78	1.00	0.06	1.69	1.55	1.84	<0.0001

AOR: Adjusted odds ratio. Ref.: Reference. ^†^ Adjusted for pre-pregnancy BMI, gestational weight gain, maternal age, race/ethnicity, marital status, education level, income level, gestational weeks, smoking status, WIC (women, infants, and children) participation during pregnancy, parity, timing of initiation of prenatal care, and number of prenatal care visits. ^‡^ Adjusted for previous history of preterm birth, pre-pregnancy BMI, gestational weight gain, maternal age, race/ethnicity, marital status, education level, income level, gestational weeks, smoking status, WIC (women, infants, and children) participation during pregnancy, parity, timing of initiation of prenatal care, and number of prenatal care visits. ^1^ The APNCU index comprises two parts: the month in which prenatal care is initiated and the number of visits from the initiation of care until delivery. Inadequate utilization is defined as either starting prenatal care after the 4th month of pregnancy or receiving fewer than 50% of the expected visits based on the schedule for prenatal care visits recommended by the American College of Obstetricians and Gynecologists (ACOG). Intermediate care is care begun by month 4 and with 50–79% of expected visits received; adequate care is that begun by month 4 and with 80–109% of the expected visits received; adequate-plus care is begun by month 4 and with 110% or more of the expected visits received.

**Table 5:** Associations of the timing of the initiation of prenatal care with small-for-gestational-age (SGA) infants and preterm birth.

	The Timing of the Initiation of Prenatal Care								
	Start Prenatal Care in the 1st Trimester	Start Prenatal Care in the 2nd or 3rd Trimester				No Prenatal Care			
	OR	AOR	95% CI		*p* Value	AOR	95% CI		*p* Value
SGA ^†^	1.00 (Ref.)	1.03	0.97	1.10	0.35	1.37	1.03	1.84	0.03
Preterm Birth ^‡^	1.00 (Ref.)	0.89	0.81	0.99	0.02	0.61	0.36	1.04	0.07

AOR: Adjusted odds ratio. Ref.: Reference. ^†^ Adjusted for pre-pregnancy BMI, gestational weight gain, maternal age, race/ethnicity, marital status, education level, income level, gestational weeks, smoking status, WIC (women, infants, and children) participation during pregnancy, parity, number of prenatal care visits, and Adequacy of Prenatal Care Utilization (APNCU) index. ^‡^ Adjusted for previous history of preterm birth, pre-pregnancy BMI, gestational weight gain, maternal age, race/ethnicity, marital status, education level, income level, gestational weeks, smoking status, WIC (women, infants, and children) participation during pregnancy, parity, number of prenatal care visits, and APNCU index.

**Table 6:** Associations of the number of prenatal care visits with small-for-gestational-age (SGA) infants and preterm birth.

	The Number of Prenatal Care Visits								
	≥12 Times	9–11 Times				≤8 Times			
	OR	AOR	95% CI		*p* Value	AOR	95% CI		*p* Value
SGA ^†^	1.00 (Ref.)	1.07	1.02	1.14	0.01	1.08	0.99	1.19	0.08
Preterm Birth ^‡^	1.00 (Ref.)	1.06	0.97	1.16	0.17	1.29	1.13	1.48	0.0002

AOR: Adjusted odds ratio. Ref.: Reference. ^†^ Adjusted for pre-pregnancy BMI, gestational weight gain, maternal age, race/ethnicity, marital status, education level, income level, gestational weeks, smoking status, WIC (Women, Infants, and Children) participation during pregnancy, parity, timing of initiation of prenatal care visits, and Adequacy of Prenatal Care Utilization (APNCU) index. ^‡^ Adjusted for previous history of preterm birth, pre-pregnancy BMI, gestational weight gain, maternal age, race/ethnicity, marital status, education level, income level, gestational weeks, smoking status, WIC (Women, Infants, and Children) participation during pregnancy, parity, timing of initiation of prenatal care visits, and APNCU index.

## References

[1] MacDorman M.F., Mathews T. (2008). Recent Trends in Infant Mortality in the United States.

[2] MacDorman M.F. (2011). Race and ethnic disparities in fetal mortality, preterm birth, and infant mortality in the United States: An overview. Seminars in Perinatology.

[3] Goldenberg R.L., Culhane J.F., Iams J.D., Romero R. (2008). Epidemiology and causes of preterm birth. Lancet.

[4] Eichenwald E.C., Stark A.R. (2008). Management and outcomes of very low birth weight. N. Engl. J. Med..

[5] US Department of Health and Human Services Healthy People 2020. https://www.healthypeople.gov/2020/topics-objectives/topic/maternal-infant-and-child-health/objectives.

[6] Vintzileos A.M., Ananth C.V., Smulian J.C., Scorza W.E., Knuppel R.A. (2002). The impact of prenatal care in the United States on preterm births in the presence and absence of antenatal high-risk conditions. Am. J. Obstet. Gynecol..

[7] Kotelchuck M. (1994). The adequacy of prenatal care utilization index: Its US distribution and association with low birthweight. Am. J. Public Health.

[8] Heaman M.I., Newburn-Cook C.V., Green C.G., Elliott L.J., Helewa M.E. (2008). Inadequate prenatal care and its association with adverse pregnancy outcomes: A comparison of indices. BMC Pregnancy Childbirth.

[9] Koroukian S.M., Rimm A.A. (2002). The adequacy of prenatal care utilization (APNCU) index to study low birth weight: Is the index biased?. J. Clin. Epidemiol..

[10] Kotelchuck M. (1994). An evaluation of the kessner adequacy of prenatal care index and a proposed adequacy of prenatal care utilization index. Am. J. Public Health.

[11] Sahu J.P., Mishra N. (2017). Correlations of APNCU index and fetomaternal out come in tribal area of chhattisgarh. Int. J. Multidiscip. Curr. Res..

[12] Alexander G.R., Korenbrot C.C. (1995). The role of prenatal care in preventing low birth weight. Future Child..

[13] VanderWeele T.J., Lantos J.D., Siddique J., Lauderdale D.S. (2009). A comparison of four prenatal care indices in birth outcome models: Comparable results for predicting small-for-gestational-age outcome but different results for preterm birth or infant mortality. J. Clin. Epidemiol..

[14] Ickovics J.R., Kershaw T.S., Westdahl C., Magriples U., Massey Z., Reynolds H., Rising S.S. (2007). Group prenatal care and perinatal outcomes: A randomized controlled trial. Obstet. Gynecol..

[15] Debiec K.E., Paul K.J., Mitchell C.M., Hitti J.E. (2010). Inadequate prenatal care and risk of preterm delivery among adolescents: A retrospective study over 10 years. Am. J. Obstet. Gynecol..

[16] Fiscella K. (1995). Does prenatal care improve birth outcomes? A critical review. Obstet. Gynecol..

[17] Lu M.C., Tache V., Alexander G., Kotelchuck M., Halfon N. (2003). Preventing low birth weight: Is prenatal care the answer?. J. Matern. Fetal Neonatal Med..

[18] Alexander G., Cornely D.A. (1986). Prenatal care utilization: Its measurement and relationship to pregnancy outcome. Am. J. Prev. Med..

[19] White D.E., Fraser-Lee N.J., Tough S., Newburn-Cook C.V. (2006). The content of prenatal care and its relationship to preterm birth in Alberta, Canada. Health Care Women Int..

[20] Ahluwalia I.B., Morrow B., D’Angelo D., Li R. (2012). Maternity care practices and breastfeeding experiences of women in different racial and ethnic groups: Pregnancy risk assessment and monitoring system (PRAMS). Matern. Child Health J..

[21] Nunes A.P., Phipps M.G. (2013). Postpartum depression in adolescent and adult mothers: Comparing prenatal risk factors and predictive models. Matern. Child Health J..

[22] American Academy of Pediatrics and American College of Obstetricians and Gynecologists (1992). Guidelines for Perinatal Care.

[23] Krueger P.M., Scholl T.O. (2000). Adequacy of prenatal care and pregnancy outcome. J. Am. Osteopath Assoc..

[24] Partridge S., Balayla J., Holcroft C.A., Abenhaim H.A. (2012). Inadequate prenatal care utilization and risks of infant mortality and poor birth outcome: A retrospective analysis of 28,729,765 US deliveries over 8 years. Am. J. Perinatol..

[25] McGregor J.A., French J.I., Parker R., Draper D., Patterson E., Jones W., Thorsgard K., McFee J. (1995). Prevention of premature birth by screening and treatment for common genital tract infections: Results of a prospective controlled evaluation. Am. J. Obstet. Gynecol..

[26] Schulte J., Dominguez K., Sukalac T., Bohannon B., Fowler M.G. (2007). Declines in low birth weight and preterm birth among infants who were born to HIV-infected women during an era of increased use of maternal antiretroviral drugs: Pediatric spectrum of HIV disease, 1989–2004. Pediatrics.

[27] Su L.-L., Chong Y.-S., Chan Y.-H., Chan Y.-S., Fok D., Tun K.-T., Ng F.S., Rauff M. (2007). Antenatal education and postnatal support strategies for improving rates of exclusive breast feeding: Randomised controlled trial. BMJ.

[28] Delvaux T., Buekens P., Godin I., Boutsen M. (2001). Barriers to prenatal care in Europe. Am. J. Prev. Med..

